# Tenofovir decrease hepatocellular carcinoma recurrence in chronic hepatitis B patients after liver resection

**DOI:** 10.1186/s13027-018-0191-8

**Published:** 2018-06-08

**Authors:** Min Zhang, Dexin Wang, Haidong Liu, Hui Li

**Affiliations:** 1 Department of Gastroenterology, Qingdao NO.6 People’s Hospital, Qingdao, Shandong 266033 People’s Republic of China; 2 10th Department of Hepatology, Qingdao NO.6 People’s Hospital, Qingdao, Shandong 266033 People’s Republic of China; 3 Invasive Technology Department, Jining NO.1 People’s Hospital, Jining, Shandong 272100 People’s Republic of China

**Keywords:** Tenofovir, Entecavir, Chronic hepatits B, Hepatocellular carcinoma, Nucleus(t)ide analogues

## Abstract

**Background:**

Tenofovir disoproxil fumarate (TDF) and entecavir (ETV) are recommended as the first-line choices regarding the treatment of chronic hepatits B. The impact of the two antiviral agents on prognosis of Chronic hepatitis B (CHB) related hepatocellular carcinoma (HCC) remains to be explored. We aim to investigate whether CHB-related HCC patients receiving TDF and ETV have a different prognosis.

**Methods:**

233 CHB-related compensated cirrhosis patients were divided into groups according to the nucleut(s)ide patients received. The results of TDF and ETV groups were reviewed and compared. The disease-free survival (DFS) and overall survival (OS) of both groups were analyzed and compared.

**Results:**

233 CHB-related compensated cirrhosis patients from 2013 October to 2014 November were included in our study. 107 and 126 patients received TDF and ETV monotherapy, respectively. Child-Pugh score, alanine aminotransferase (ALT), aspartate aminotransferase (AST), total bilirubin level, status of hepatitis B e antigen (HBeAg) and serum HBV DNA level were compared between groups. DFS in TDF-treatment group were significantly longer than it in ETV-treatment group (*p* < 0.05). multi-variant analysis indicated that TDF duration was significantly associated with lower probability of HCC development, (hazard ratio, 0.35; 95% confidence interval range, 0.33–0.84, *p* < 0.05).

**Conclusion:**

Anti-virus regimen containing TDF benefits for the prognosis of CHB-related liver cirrhosis patients.

## Background

Hepatocellular carcinoma (HCC) is among the most common causes of cancer-related deaths with a high prevalance worldwidely, [1] of which Chinese patients acounts for over 50%. [2, 3] For the high incidence of recurrence, the prognosis and disease-free survival of patients after curative resection remain to be improved, although surveilance of patients with chronic hepatic diseases has been enhanced. [4–6] Advances of HCC recurrence have revealed a number of risk factors, including the serum α-fetoprotein (AFP), tumor stage, cirrhosis, chronic hepatits B and heptitis C. [5]

More than 50% incidence of HCC is associated with chronic hepatits B virus (HBV) infection. [7, 8] In China, about 100 million people are infected with HBV and 20% of them will progress to chronic infection. [9, 10] 10~ 20% of patients will develop cirrhosis within 5 years. [11] High HBV viral load has been determined to be associated with tumor relapse in HBV-related HCC. [12, 13] As one of HCC risk factors, HBV viral load can be controllable with effective antiviral agents such as tenofovir disoproxil fumarate (TDF) and entecavir (ETV), [14, 15] which are recommended as the first-line therapy by international anti-HBV guidelines. [16–18] Long term duration of nucleus(t)ide analogues (NAs) to continuous suppress relication of HBV has been proved to be associated with regression of cirrhosis and decompensated hepatic diseases. [19, 20] Evidences also indicated NAs reduce the risk of CHB-related HCC development. [21–24]

TDF and ETV were determined to have high barrier to clinical resisitance and better viral response rate. [14, 15] But whether there are discrepancies of the two first-line antiviral agents regarding the prognosis of CHB-related HCC patients after surgeries remains to be explored. Recent advance indicated that nucleutide analogues, rather than nucleuside analogues can induce the expression of IFN-λ, which might be another target for the antiviral therapy. [25] We suppose the induction of IFN-λ expression may also exert impact on the prognosis of CHB-related HCC patients. We conduct a retrospective study to review and compare the survival of CHB-related HCC patients after curative liver resection.

## Methods

### Study population

All the patients received liver resections between 2013 October to 2014 November at Jining NO.1 People’s Hospital (Shandong, China). The anti-viral treatment were conducted at Qingdao NO.6 People’s Hospital (Shandong, China). All the clinical data were retrieved from hospital electronic database. Patients accepted anti-viral treatment at least for 6 months before they underwent surgery. We excluded patients with concurrent viral infections, such as hepatits C, hepatitis D and human immunodeficiency virus, Child-Turcotte-Pugh (CTP) scoring ≤9,alcoholic hepatic diseases, invalid clinical characteristics and laboratory outcomes and patients received TDF/ETV combination therapy or antiviral regimen switch from TDF to ETV during follow-up, and vice versa. Overall, the initial cohort consisted of 321 patients, 85 patients were excluded for co-infected with HCV (*n* = 21) and HDV (*n* = 3), regimen switch (*n* = 37) and invalid clinical data (*n* = 27). The final patients included were 233.

This study was conducted under compliance with the Declaration of Helsinki and obtained by *the Human Ethics Committee of Jining NO.1 People’s Hospital and the Human Ethics Committee of Qingdao NO.6 People’s Hospital.*

### Antiviral treatment and follow-up

HBV infection were diagnosed with positive serum viral marker and elevated serum HBV-DNA level (> 1000 copies/mL during two consecutive detection). Serum HBV DNA level was quantified by real-time quantitative PCR assay with Roche LightCycler (Roche Diagnostics, Basel, Switzerland) and suitable reagents (PG Biotech, Shenzhen, China), the lower limit of quantification is 1000 copies/mL and the linear range was between 1120 and 6.69 log copies/mL. HCC and cirrhosis were histologically confirmed through specimen from sugeries. Contrast-enhanced CT, ultrasonography or liver biopsy were conducted to screen HCC recurrence during follow-up. Child-Pugh scoring was applied for consideration of prognosis as previously reported. 107 patients received TDF monotherapy, of which 65 patients were switch from lamivudine monotherapy or lamivudine in combination with adefovir. 126 patients received ETV monotherapy, of which 81 patients were switch from lamivudine monotherapy or lamivudine in combination with adefovir. Total follow-up period of all patients ranged from 6 months to 41 months and the median period of follow-up is 28 months. Patients underwent follow-up examination at least every 6 months, including serum AFP level, contrast-enhanced and serum viral load.

### Statistics

Continuous variables were expressed as mean ± SD with normal distribution and median (range) without normal distribution. The comparison of continuous variables with or without normal distribution was analyzed with Student *t* test and Wilcoxon rank test, respectively. Chi-square and Fisher’s test were applicated for analysis of categorical variables. *P* < 0.05 was regarded as statistically significant. The univariate analysis of factors associated with overall survival of patients were conducted through Kaplan-Meier statistics and Log-rank test. Multivariate analysis was assessed with Cox regression test. Variables with *p* < 0.05 were employed into the Cox regression model. *P* < 0.05 was considered as statistically significant. Statistics analysis was conducted with SPSS (version 16.0, SPSS Inc., Chicago, IL, USA) software package. Figures were made with GraphPad Prism 5 software.

## Results

### Baseline characteristics

The baseline characteristics were presented in Table 1. The mean age of patients is 54 years old. Ranging from 25 to 73. Most patients were male (*n* = 182, 78.11%). The CTP scoring of patients were predominantly A (*n* = 227, 97.42%) and no patients were classified as CTP C scoring. The average level of serum viral load was 3.9 log copies/mL, ranging from less than 3 to 5.1 log copies/mL and 70.39% patients were positive for HBV e antigen. 96 patients were histologically confirmed with cirrhosis. Median size of tumor was 4.1 cm, ranging from 2.1 to 9.7. The average total bilirubin level is 5.87 mmol/L, 15 patients had jaundice caused by tumor invasion of hepatic bile ducts and recovered to normal soon after surgery.

**Table 1:** Baseline characteristics

Variable	Value	
Patients, *n*	233	
Male sex, *n* (%)	182	(78.11)
Age, M (range)	54	(25–73)
CTP class A/B/C, *n* (%)	227**/**6**/**0	(97.42**/**2.58**/**0)
Tumor maximum size, cm, M (range)	4.1	(2.1–9.7)
HBV DNA, log copies/ml	3.9	(3.0–5.1)
HBeAg postive, *n* (%)	164	(70.39)
Cirrhosis, *n* (%)	96	(41.20)
AFP, ng/mL, M (range)	103.7	(7.8–1210)
TBIL, mmol/L, M (range)	5.87	(2.60–64.70)
ALT, IU/L, M (range)	26	(11–57)
AST, IU/L, M (range)	27	(19–78)
ALP, IU/L, M (range)	51	(45–187)
ALB, g/L, M (range)	3.9	(2.3–5.7)
PLT, 10^9/L, M (range)	163.5	(79–241)
PT, s, M (range)	12.1	(11.3–13.6)
INR, M (range)	0.99	(0.77–1.24)

AFP = α-fetoprotein, ALB = albumin, ALP = alkaline phosphatase, ALT = alanine aminotransferase, AST = aspartate aminotransferase, CTP=Child–Trucott–Pugh score, HBV = hepatitis B virus, INR = international normalized ratio, PLT = platelet, PT = prothrombin time, TBIL = total bilirubin

Patients were divided into groups regarding their antiviral regimen after liver resections. 107 patients received TDF monotherapy and 126 patients received ETV monotherapy. The median serum AFP level of TDF group and ETV group were 97.5 and 109 ng/mL, respectively. The number of patients (*n* = 59) with cirrhosis in TDF group were significantly larger than patients (*n* = 37) in ETV group (*p* < 0.05). 21% patients in TDF group and 28% patients in ETV group were positvie for serum HBV DNA (> 1000 copies/mL). The other clinical parameters were comparable between two groups, including CTP scoring, serum viral load, albumin, alkaline phosphatase (ALP), alanine aminotransferase (ALT), aspartate aminotransferase (AST) and platelet count (Table 2).

**Table 2:** Comparison of baseline variables in patients receiving TDF and ETV

Variable	TDF		ETV		*P*
Patients, *n* (%)	107	(45.92)	126	(54.08)	
Male sex, *n* (%)	82	(76.63)	107	(84.92)	0.08
Age, M (range)	52	(25–69)	55	(26–73)	0.31
CTP class A/B/C, n (%)					0.11
A	105	(98.13)	122	(96.82)	
B	2	(1.87)	4	(3.18)	
C	0		0		
HBV DNA, log copies/ml	3.7	(3.0–4.7)	4.1	(3.0–5.1)	0.07
HBeAg postive, *n* (%)	76	(71.03)	88	(69.84)	0.14
Cirrhosis, *n* (%)	59	(55.14)	37	(29.36)	0.03
AFP, ng/mL, M (range)	97.5	(7.8–1210)	109	(10.3–1210)	0.33
Tumor maximum size, cm, M (range)	3.8	(2.8–9.7)	4.4	(2.6–8.5)	
TBIL, mmol/L, M (range)	4.78	(2.6–64.7)	7.41	(3.90–54.30)	0.16
ALT, IU/L, M (range)	21	(11–41)	27	(19–57)	0.11
AST, IU/L, M (range)	28	(19–56)	21	(20–78)	0.41
ALP, IU/L, M (range)	49	(45–111)	58	(51–187)	0.09
ALB, g/L, M (range)	3.5	(2.3–5.7)	4.1	(2.5–5.1)	0.64
PLT, 10^9/L, M (range)	134.24	(79–241)	183	(99–210)	0.21
PT, s, M (range)	11.9	(11.3–12.6)	12.5	(11.7–13.6)	0.08
INR, M (range)	1.01	(0.77–1.24)	0.97	(0.81–1.21)	0.51

AFP = α-fetoprotein, ALB = albumin, ALP = alkaline phosphatase,ALT = alanine aminotransferase, AST = aspartate aminotransferase, CTP=Child–Trucott–Pugh score, HBV = hepatitis B virus, INR = international normalized ratio, PLT = platelet, PT = prothrombin time, TBIL = total bilirubin

### Virological response and serum biomarker dynamics

of 233 patients, 132 patients were NAs experienced (NAs duration > 12 months) and 101 patients were NAs naïve. No patients received interferon for anti-HBV treatment. The median duration of TDF and ETV were 24 and 26 months, respectively. All patients were serum viral load negative after 12 months NAs treatment and no patients relapsed during follow-up period.

In TDF group, 21 patients were NAs naïve and 86 were NAs experienced; In ETV group, the number was consists of 80 NAs naïve patients and 46 NAs experienced patients. 21 patients in TDF group and 31 patients in ETV group experienced viral breakthroughs (> 1000 copies/mL during two consecutive detection) for at least once, all of which were related to poor adherence. We checked the adherence of patients by judging whether their pills matched the days between two administrations of NAs.

During the follow-up period, 68 of 164 HBeAg postive patients (41.46%) had HBeAg disappeared and 31 patients (18.90%) had HBeAg seroconversion. No HBsAg disappearance or seroconversion was observed.

### Survival analysis

**Fig. 1:**
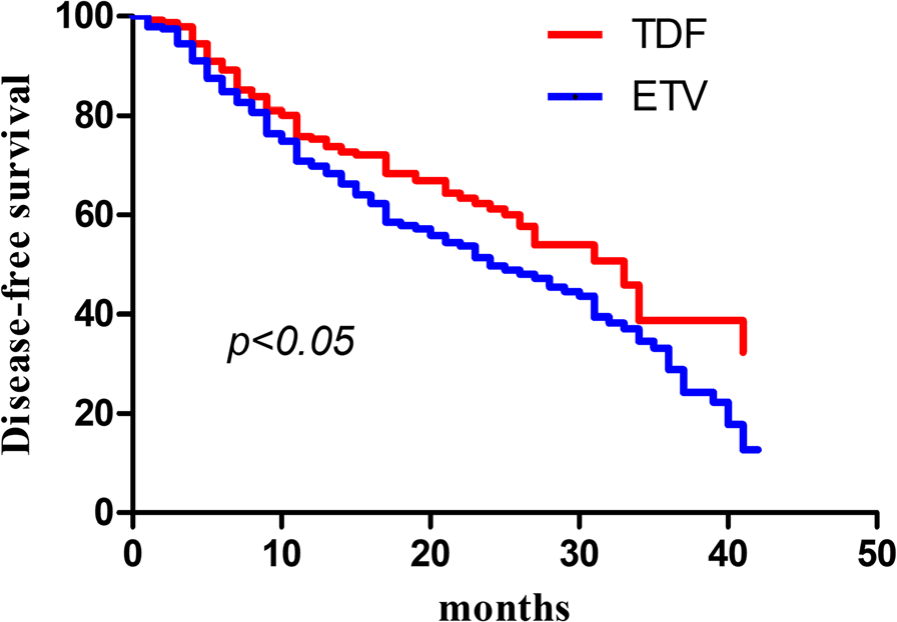
The disease-free survival of CHB-related HCC patients after surgery. The comparison of cumulative HCC development probability between TDF group (red) and ETV group (blue). X-axis represented time (month), Y-axis represented disease-free survival

During the follow-up period, 174 (74.68%) patients experienced HCC recurrence and 47 patients died. In order to compare the difference between TDF and ETV group, we conducted survival analysis with Kaplan Meier curve. The median DFS of TDF and ETV group were 33 and 24 months, respectively. The results showed that disease-free survival (DFS) of TDF group was significantly longer than ETV group (*p* < 0.05). (Fig. 1).

### Predictors for disease-free survival

In order to verify the potential effect of TDF treatment duration on disease-free survival of CHB-related HCC patients. We conduct both univariant and multi-variant analysis to potential factors that might associated with DFS. The results of univariant analysis showed that status of HBeAg (hazard ratio, 0.61; 95% confidence interval, 0.11–0.91; *p* < 0.05), non-cirrhosis (0.47, 0.21–0.98, *p* < 0.05) and TDF duration (0.78, 0.43–0.97, *p* < 0.05) were associated with disease-free survival. Further multi-variant analysis using Cox regression model indicated that TDF treatment duration (0.35, 0.33–0.84, *p* < 0.05) and non-cirrhosis (0.41, 0.13–0.77, *p* < 0.05) were independently associated with better disease-free survival.(Table 3).

**Table 3:** Univariate and multivariate analysis of disease-free survival

	Univariant analysis	*p*-value	Multivariant analysis	*p*-value
	HR (95% CI)		HR (95% CI)	
Gender:male/female	0.77 (0.37–1.59)	NS		
Child-Pugh score: A/B	0.94 (0.32–1.61)	NS		
HBV DNA ≤ 4 (log copies/mL)	0.71 (0.33–1.54)	NS		
HBeAg: negative/positive	0.61 (0.11–0.91)	< 0.05		
Non-cirrhosis/Cirrhosis	0.47 (0.21–0.98)	< 0.05	0.41 (0.13–0.77)	< 0.05
Total bilirubin: < 24/≥ 24 (μmol/L)	1.15 (0.69–1.91)	NS		
TDF treatment/ETV treatment	0.78 (0.43–0.97)	< 0.05	0.35 (0.33–0.84)	< 0.05

AFP = α-fetoprotein; HBeAg = hepatitis B e antigen; HBV = hepatitis B virus

## Discussion

Active HBV replication is significantly associated with the recurrence of hepatocellular carcinoma after surgery. [6] Although continuous supression of HBV has been proved to be effective in reducing the incidence and recurrence of HCC with solid evidence. [26, 27] Both ETV and TDF were recommended as first line agents for anti-HBV therapy and regarded with high efficiency and high barrier to genetic resistance. [14, 15] Recent clinical evidence showed that up to 10% of patients with HBV can develop HCC even with effective anti-virus agents. [28] Our study showed that CHB-related HCC patients received TDF after surgery have better DFS, compared to patients received ETV. The underlying mechanism might be complicated. However, recent advance showed that nucleutide analogues, rather than nucleuside analogues can induce the expression of IFN-λ [25]. since interferon-λ3 has been demonstrated to be involved in modulation of imunity during virus infection or autoimune diseases [29]. Inflammation is determined to have a strong association with carcinogenesis and recurrence of HCC [30]. Thus, we supposed that ADV might regulate the immunity through induction of interferon-λ3 to improve the survival of CHB-related HCC patients in our study. However, it requires further studies to prove our hypothesis.

Sustained suppression of HBV replication is critical for lowering recurrence incidence of CHB-related HCC patients after surgery. [27, 28] Since most patients receiving their anti-HBV treatment out-of-hospital, it’s difficult for us to conduct close monitoring on their adherence. 52 patients (22.32%) experienced transient serum viral load fluctuation due to poor adherence. Regarding the actual obstacle of monitoring, we develop an easy and practical method by matching the days between two consecutive administration date and number of pills remained to help patients self-monitor drug adherence. The serum viral load of patients experienced viral breakthoughs returned to negative after continuous administraion. We compared the DFS between patients with viral breakthrough and patient without, the results showed there was no significant difference between two groups.

We include patients both naïve and experienced to NAs and we also compared the potential difference. However, only the number of patients experienced viral breakthrough in NAs experienced group was higher than that of patients naïve to NAs (41 vs. 11). Due to our retrospective design and population included, further study with prospective attempt and de novo comparison may provide a better understanding regarding patients receiving different NAs both in single and in combination. And due to the financial situation of patients included, consecutive monitoring on the quantification of serum viral markers, such as HBsAg, was limited, which might provide a clue to explore the potential mechanism.

## Conclusions

In conclusion, our study suggested the different impact of TDF and ETV on prognosis of CHB-related HCC patients. Patients receiving longterm TDF treatment had a longer DFS than patients receiving ETV. We shall confirm these results with prospecitve study and lager sample size.

## Abbreviations

ALT: Alanine aminotransferase
CHB: Chronic hepatits B
ETV: Entecavir
HBeAg: Hepatitis B e antigen
HBsAg: Hepatits B surface antigen
HBV: Hepatitis B virus
HCC: Hepatocellular carcinoma
LAM: Lamivudine
LdT: Telbivudine
NAs: Nucleus(t)ide analogues
SD: Standard deviation
TDF: Tenofovir disoproxil fumarate
